# Supplementary material for: Neighborhood satisfaction and reproductive status
Source: PLoS One. 2022 Sep 7;17(9):e0273082. doi: 10.1371/journal.pone.0273082 (PMC9451072; doi:10.1371/journal.pone.0273082)
Supplement: Attachment — Submitted filename: Letter2.pdf [file pone.0273082.s001.pdf]

Summary of revisions to:
"Neighborhood satisfaction and reproductive status" (PONE-D-22-11374)

Dear Dr. Carswell and Anonymous Reviewers,

Thank you for these very helpful comments, and for the opportunity to revise and resubmit. We have revised our manuscript to address your concerns, and as a result, we believe the new version of the manuscript is improved. In this letter, we include point-by-point abridged versions of each of the reviewers' comments (in **bold**) followed by an explanation of our response. Changes in the manuscript are noted using **bold red** text.

1 Reviewer #1

1.1 "This manuscript uses survey data to focus on the question of whether neighborhood satisfaction differs among persons based on their status as a household with children. It uses multiple categories to capture this construct in novel ways, and demonstrates heterogeneity across these groups in neighborhood satisfaction. This is a well-done study, and I only have a small number of comments."

We're happy to hear you found the study well-done, and appreciate your helpful comments!

1.2 "For the SWLS measure, it would be good to list all the questions that are part of this scale."

In the methods section, we now list all five items on the SWLS, which is the most widely used scale for measuring life satisfaction.

1.3 "You do have a relatively small amount of missing data. Nonetheless, it would likely be better to use multiple imputation to address missingness, rather than listwise deletion. It likely will not change the results much, but would be more appropriate."

Thank you for this suggestion. We have elected not to perform multiple imputation for two reasons. First, we used Little's Test (Little, 1988, https://doi.org/10.2307/2290157) to test whether our data are missing completely at random (MCAR). This test ($\chi^2_{44} = 51.54$, $p = 0.203$) indicates the data is MCAR, and therefore listwise deletion does not introduce any bias. Second, our analysis uses survey sampling weights to ensure representativeness and allow us to make population inferences, but methods of multiple imputation with sampling weights are not well developed. Ignoring the survey weights for the sake of using multiple imputation would introduce biases.

1.4 "I appreciated Table 4, and showing the statistical significance between different categories."

We're glad this was helpful, and have kept it in this revision.

1.5 "In section 5.2 you hypothesize that SES may be a possible explanation. It appears that this is not included in the model? It would be reasonable to include this to assess this possible explanation."

Socio-economic status is often measured using education, income, or a combination of the two. In this analysis we include education as a measure of SES. Unfortunately, these data do not include a usable measure of income; the response options were incorrectly programmed by IPPSR at the time they designed the survey. Therefore, we are unable to control for income.

2 Reviewer #2

2.1 "This paper focuses on parental status as having a significant effect on neighborhood satisfaction. The authors find that single parents and childfree individuals experience significantly less neighborhood satisfaction than other groups. The paper explores the nuances of understanding neighborhood satisfaction and adds an often missing area of extant literature."

We're happy to hear that you found out work addresses an area that is currently missing from the literature.

2.2 "Reproductive Statuses – I agree with the sentiments and framing of this identification or state of being but maybe considering another term like "parental statuses" (as noted in the paper) or "familial statuses" as 'reproductive statuses' embodies some assumptions around actual reproductive abilities. That is, categories such as "not-yet-parents" infers that people have the ability to be parents in the future."

We appreciate this observation and feedback, and agree that it's difficult to know what the best terms are. Because these are the terms and classification used in prior studies (Neal & Neal 2021, 2022), we have elected to retain them here for consistency. Early in our research we considered the term 'parental status,' but felt that it foregrounded parents over other groups. We also considered 'familial status,' but were concerned that it could easily be confused for the construct of relationship status (e.g., single, married, divorced, widowed, etc.).

2.3 "Data – Which version of the ACS are the authors using in their analysis? It says 2016 ACS but is that the one, three, or five year estimates? Moreover, are these households clustered in any particular region or spatially distributed across the state, in urban, suburban and more rural spaces?"

We have clarified that the Institute for Public Policy and Social Research (not us) used 1-year ACS estimates to compute sampling weights for these data. The sampling weights were not designed to ensure representativeness by geographic area; this would have required a dramatically larger sample. However, we have clarified that "Respondents were recruited throughout the state of Michigan, including from rural and urban areas, with counties represented roughly proportionally to their population size."

2.4 "Timing of Data Collection – Were there any other sociopolitical events worth noting during the data collection time in Michigan? The governor kidnapping plot comes into mind but that was after the data collection period."

You are correct that news of the plot to kidnap Governor Whitmer became public in October 2020, several months after data collection in May 2020. However, in the 'data' subsection we comment on two other important events: "Data collection occurred during the initial wave of the COVID-19 pandemic when residents were under a stay-at-home order from the Governor. Data collection ended on the day that George Floyd was murdered by a police officer in Minneapolis, resulting in widespread protests against police brutality and systemic racism. Based on the survey's timing, respondents' reported neighborhood satisfaction may have been affected by the COVID-19 pandemic, but was not influenced by the murder of George Floyd or subsequent protests."

2.5 "Covariates – The authors make several methodological choices which need further clarification. For example, for racial categories, the study includes White and Persons of Color. Given that this is a representative sample with almost 77% of it being White households, are diverse are racial and ethnic categories among households of color? Moreover, given that the percentage of White households is much higher than the state of Michigan (approx. 62% in 2021), is there any potential biases introduced into the analysis?"

Thank you for pushing us to clarify this important issue. We have clarified that race is coded into a binary variable distinguishing 'white alone, not hispanic' from all other respondents. Unfortunately there are too few respondents in any other single racial or ethnic group to permit stable estimates using a finer grained coding. This is the coding scheme often used by the U.S. Census when reporting race and ethnicity. As we report in Table 1, our weighted data estimate that 76.8% of the population is white alone, not hispanic. This closely matches the U.S. Census 2021 Population Estimates Program estimate of 74.2% (see `https://www.census.gov/quickfacts/MI`). Therefore, we believe our sample (particularly when analyzed with sampling weights) is representative, and biases by race are unlikely.

2.6 "Moreover, income was not controlled for. Is this sample reflective of all households or particular income brackets? Other studies tend to focus on particular types of neighborhoods (such as low-income neighborhoods (See Ciorici and Dantzler 2019 or Jones and Dantzler 2021) or in localities undergoing significant redevelopment or relocation efforts (See Oakley et al. 2013). Given the framing of the paper, particularly the disaggregation of parental statuses, it seems that other coding choices should follow suit. Yet, the cross-sectional nature of the data may prohibit further disaggregation."

Unfortunately, these data do not include a usable measure of income; the response options were incorrectly programmed by IPPSR at the time they designed the survey. Therefore, we are unable to directly control for income. However, the sampling weights were computed to ensure the sample is representative with respect to income. Additionally, we do control for education, which is typically highly correlated with income. We also identify the inability to compare different types of neighborhoods as a limitation, and point readers to recent work by Dantzler in this vein as a future direction.

Due to confidentiality, the data provided by IPPSR does not contain detailed geolocations for respondents. Therefore, we are unable to restrict the focus to particular types of neighborhoods, or to compare residents living in different types of neighborhoods.

2.7 "And lastly, what about housing tenure statuses? Do you have data on whether households are renting versus owning? In addition, is there data on their specific type of housing (i.e., apartment versus single family home)? Seems like these are important measures to consider given the diversity of neighborhoods surveyed in this study. Should none of these variables be available, the authors may want to add a couple sentences in their limitation section to address this."

Measures of housing type or tenure are not available in these data. We now note this as a limitation.

2.8 "Analyses – When including the covariates, the authors note that they may be association with reproductive statuses and neighborhood satisfaction. To control for the possible endogeneity here, have the authors considered other methodological approaches such as employing an SEM or HLM approach? While the analyses here is straightforward, I worry that some of the possible endogeneity imputed from this approach may be driving some of these estimates in particular ways."

A structural equation model (SEM) would be appropriate if we were investigating the role of one or more latent factors. However, because all our variables are measured, as opposed to latent, we do not believe SEM is appropriate.

A hierarchical linear model (HLM) or mixed model would be appropriate if neighborhood satisfaction was clustered by higher-level (e.g., tracts, neighborhoods) units, or if our analysis included covariates measured at a higher level (e.g., tract demographic composition). We do not pursue an HLM for two reasons. First, a recent meta-analysis of 27 studies found that there is little clustering of neighborhood satisfaction by areal units such as neighborhoods, tracts, or streets (Neal, 2021, `https://doi.org/10.1177/0042098020926091`). Second, these data and our models do not contain information about respondents' geographic locations that could be used to measure higher-level covariates or to define a level-2 equation in an HLM.

2.9 "Overall, I found the paper theoretically interesting and methodologically sound. However, I am offering a Revision for the purposes of addressing my earlier comments and critiques. I look forward to seeing a revised version of the paper."

We are happy to hear that you found the paper both interesting and methodologically sound. As we note above, we have been able to directly incorporate many of your suggestions directly into the revision. For those that we were unable to incorporate, mainly due to the lack of suitable data, we now discuss them as limitations and directions for future research.
